# Working with a youth mental health apprenticeship scheme to coproduce evidence synthesis: The youth mental health evidence synthesis hub

**DOI:** 10.1002/jcv2.70094

**Published:** 2026-01-11

**Authors:** Ruth Wadman, Olivia Taylor, Chloe Parekh, Rachel Churchill

**Affiliations:** ^1^ Department of Health Sciences The University of York York UK; ^2^ Department of Psychology University of Bradford Bradford UK; ^3^ Bradford Institute for Health Research Bradford UK; ^4^ Centre for Reviews and Dissemination The University of York York UK

**Keywords:** coproduction, evidence synthesis, PPI, young people, youth mental health

## Abstract

**Background:**

Meaningful involvement of young people in mental health research ensures that it is relevant and has impact. Traditional approaches to patient and public involvement often fail to provide sustainable and reciprocal engagement. We reflect on our experiences of working with an apprenticeship model as an alternative approach to involving young people in mental health research.

**Methods:**

We describe working with the Bradford Healthy Minds Apprentices, a group of 16‐ to 24‐years‐olds engaged in a year‐long paid apprenticeship scheme. This partnership involved coproduction activities to develop the Youth Mental Health Evidence Synthesis Hub (Y‐MHESH), including workshops, website design and short videos. We focussed on relationship‐building and provided clear feedback to demonstrate how the research team had acted and adapted in response to input from the Apprentices.

**Results:**

Working with the Apprentices offered distinct benefits to both researchers and the young people, including community engagement, flexible collaboration and opportunities for future working. The Apprentices were an established and externally supported group which facilitated relationship‐building. Their professional status fostered a more equal and reciprocal partnership. The Apprentices themselves gained research skills and were able to choose their level of involvement in Y‐MHESH. Through regular feedback they felt their contributions were valued by the researchers, engendering trust. Challenges included adapting to group dynamics and preferred ways of working, limited availability, funding limitations, and unfamiliar payment processes.

**Conclusion:**

Partnerships with groups like the Healthy Minds Apprentices, who are paid and supported within a relevant organisation, can support reciprocal coproduction and community‐linked, bottom‐up research. We suggest that a similar university‐based apprenticeship model could offer a way to sustainably involve young people in mental health research, as well as providing developmental opportunities for young people. While requiring institutional buy‐in and flexibility, this approach to involving young people aligns closely with the university's commitment to public good.

## Key points

**What's known?**

- It can be difficult to achieve sustainable and meaningful youth involvement using traditional patient and public involvement models.

**What's new?**

- We worked with a group of young people employed as mental health apprentices on coproduction workshops and produce short videos for the Youth Mental Health Evidence Synthesis Hub.
- Key advantages of working with an established group employed and supported by an external organisation were relationship building and community links. We also identified ways to improve this model of working.

**What's relevant?**

- Young people had the opportunity to develop research and related skills and the chance to continue working with the research team in the future.
- A university‐based research apprentice model could support sustainable and reciprocal youth involvement in mental health research.

## INTRODUCTION

There is growing recognition of the importance of involving the young people in the research that affects them. Involving young people in research, through involvement activities and coproduction, can ensure the research is relevant and appropriate, and that the right questions are asked. In mental health research, young people are often not given adequate opportunities to shape research in this way (Larsson et al., 2018). Although patient and public involvement (PPI) in mental health research is becoming more established, young people (especially those with mental health conditions) are still overlooked. Their contributions to research can be limited by assumptions about their vulnerabilities and ability to understand, contribute to and influence research (Murray, 2005). It can also be difficult to involve young people in research in a consistent and sustainable way. This is in part due to the costs involved (Pizzo et al., 2015) but also the evolving nature of young people's lives as they move through education and into employment.

In this paper we reflect on our experiences of working with young people employed as apprentices to do PPIE activities and to coproduce mental health evidence synthesis projects. We suggest that this apprenticeship approach could be more sustainable than traditional public involvement approaches; for instance, PPI groups for specific studies, often with a limited scope and lifespan, where contributors receive one‐off payments (sometimes in shopping vouchers) for their involvement in a workshop. Proof of concept and evaluation of a similar pilot scheme to train peer researchers has been undertaken by The Young Foundation (July 2022), demonstrating that this model can be successfully implemented and is acceptable to stakeholders. We describe the benefits of this approach and also the challenges we encountered when working with young people as apprentices. We also consider potential challenges involved in adopting this apprenticeship model.

## INVOLVING YOUNG PEOPLE IN MENTAL HEALTH RESEARCH

Half of mental health disorders start by age 14 years and three fourths emerge by age 24 (Kessler et al., 2005). The prevalence of mental health difficulties in children and young people has increased over the last 20 years (Cybulski et al., 2021). In 2022, an estimated 1.4 million children and young people in England sought NHS help for mental health problems (Newlove‐Delgado et al., 2022). Despite significant research funding into child and adolescent mental health, improvements in mental health have not been realised. We need to better understand and involve young people in the research that impacts on them, to create real change to improve the mental health of society. Mental health has been identified as a top concern and priority for young people today, along with education and preparing for the future (The Young Foundation, March 2022). The peer researchers contributing to that report highlighted a lack of mental health support and a lack of career opportunities, particularly for young people living in the North of England.

Involving young people in mental health research as public and/or lived‐experience contributors has the potential to transform research, policy and practice. There is growing consensus that research that affects young people is of greater value if young people are meaningfully involved throughout the research life cycle (Kelly et al., 2018; Pavarini et al., 2019). This includes shaping topic priorities, project design and conduct, interpretation, communication and dissemination, as well as evidence syntheses that directly influence policy and practice.

The move towards coproduction in health research is welcome, as it aims to involve members of the public as equal partners in shaping research and discovering new knowledge. Equality, inclusion and redressing power imbalances between researchers and contributors are central principles of coproduction. However, this approach brings even greater challenges around resourcing and sustainability. When coproduction endeavours are not adequately resourced then there is a risk that they are experienced as tokenistic and extractive (Mawn et al., 2015). Even when adequately resourced, there is an inherent ‘messiness’ in collaborative mental health research and a considerable amount of emotional labour, which is not often recognised (The PARTNERS2 writing collective, 2020). Therefore, careful consideration of these practical and emotional demands is crucial when planning coproduction in mental health research.

There is a limited literature on research involvement in young people's mental health research (Norton, 2021), and particularly research evaluating the impact of PPI and coproduction in youth mental health (Fernandes et al., 2023). A recent systematic review looked at barriers to, and facilitators for, youth involvement in mental health research focussing on underrepresented groups (Perowne et al., 2025). This review recommended inclusive and flexible research inclusion practices with underrepresented young people and that improved reporting of youth involvement practices and strategies is also needed. We also note two published protocols outlining planned evidence syntheses; a systematic review of Patient and Public Involvement (PPI) in youth mental health research (Sales et al., 2021) and a scoping review of PPI and Responsible Research and Innovation (RRI) in mental health projects involving young people (de Alcântara Mendes et al., 2024). Overall, there is limited information about commonly used approaches and practices in PPIE and coproduction reported in the mental health literature, and there has been a recent call for researchers to report on adolescent engagement in research in a more systematic and detailed way (Lloyd et al., 2024; Nagata et al., 2025). Case studies and short reports are increasingly being used as an effective format for reporting innovative and creative developments in coproduction and involvement with young people. These descriptive and reflective accounts can make a valuable contribution to the literature, in addition to more conventional research reports.

High quality evidence synthesis in the field of child and adolescent mental health is essential. It supports reproducible research by providing a transparent and systematic approach to reviewing and integrating evidence, uncovering potential biases, and providing insights that will strengthen the rigour and relevance of future research (Bellato et al., 2023). There has been a marginal increase in PPIE in systematic reviews and other evidence syntheses in recent years, alongside recommendations that the nature and impacts of this involvement be clearly described (Abrams et al., 2021). A recent umbrella review of evidence syntheses involving adolescents found that although youth involvement to date is limited, young people can contribute meaningfully across the stages of the review process such as interpretation of findings, study selection, and data analysis (Warraitch et al., 2025). This may be especially pertinent for youth involvement in mental health evidence synthesis, where more nuanced, qualitative and complex reviews may be needed (Knowles et al., 2022). In a complex evidence synthesis of interventions to improve the mental health of young people with long‐term physical health conditions, the authors report important ways in which their young people's advisory group contributed to the study, particularly by using their lived experience to help the researchers situate their study within a wider context. This included sense checking the search terms, contributing to (and to some extent challenging) the preliminary themes in the qualitative synthesis and creating a comprehensive dissemination strategy (Walker et al., 2021). We suggest that transparent reporting of youth involvement in mental health research, including evidence synthesis, is a key step in building the evidence base.

## METHODS

### The Bradford healthy minds apprentices

The Bradford Healthy Minds Apprentices are a group of 16‐ to 24‐year‐olds working on a paid 1‐year mental health apprenticeship programme based in the community sector and supported by a specialist youth organisation. They are a group of young people who are interested in mental health, and who work across Bradford and Craven (in the North of England) to support young people, communities and schools with their health and wellbeing. As part of a broader portfolio of youth work, community action and advocacy in mental health services, this group contribute to local research projects including the Born in Bradford Age of Wonder cohort study (Ryan et al., 2024; Shire et al., 2024). The group were commissioned by Bradford District and Craven Health and Care Partnership in 2020 to help shape local mental health services work, deliver awareness workshops in the community, conduct academic reports and audits, and take part in coproduction, co‐design, community action and youth engagement. There have been five cohorts of Healthy Minds Apprentices.

We worked with the third cohort of Apprentices in 2022–2023 on an evidence synthesis coproduction project (Y‐MHESH—described below). This group included two males and four females. They were supervised by two project workers (who had completed the apprenticeship the previous year) who also took part in the coproduction activities. We were able to travel to meet with the Apprentices in their office space situated in a community centre in Bradford. Some meetings were online or hybrid. We facilitated four workshops with the group between January and July 2023 and completed additional tasks via email. An overview of the workshops is given in Table 1. In addition, the group took part in a 2‐day filming session with a video production company. An additional workshop in October 2023 was used to introduce the research team to the newly appointed Apprentices (fourth cohort) and to find out what their mental health interests were.

**TABLE 1:** Overview of Y‐MHESH workshops.

Workshop 1 (January 2023)	Introduction to evidence synthesis ‐ Look at the Cochrane Common Mental Health Disorders webpages and examples of systematic reviews looking at children and young people's mental health ‐ Discussion around things that have helped the Apprentice understand technical research terms ‐ Discussion generating ideas for the Y‐MHESH website
Interim work	Review example animations about health (e.g., epilepsy) and prepare feedback on design and format.
Workshop 2 (March 2023)	Animation explaining systematics reviews and film plans ‐ Animation script read‐through and edits from the group ‐ Overview of filming plans and agreeing draft film interview questions ‐ Finalise animation feedback to share with the video production company
Interim work	Reviewing animation style options and voice‐over actors, and choosing preferred options
Workshop 3 (May 2023)	Priority topics for evidence synthesis ‐ Hands‐on overview of how research funding operates ‐ Discussion to agree priority research topics ‐ Session reading through plain English summaries of completed systematic reviews on young people's mental health; chance to ask question about the review process
Interim work	Provide individual feedback on the draft versions of the animation and film
Workshop 4 (July 2023)	Y‐MHESH website ‐ Discussion of content/look and feedback from the group

### Youth mental health evidence synthesis hub (Y‐MHESH)

Working with the Bradford Healthy Minds Apprentices we established the Youth Mental Health Evidence Synthesis Hub (Y‐MHESH), an initiative coproducing evidence syntheses to ensure the right questions are asked about the things that are important to young people. Meaningful evidence synthesis in youth mental health should aim to involve young people themselves. The benefits of working with the young people directly in this way makes the research relevant. Involving research‐users, in this case young people, in the production of reviews and evidence synthesis is recommended to increase the likely relevance and utility of the review (Boote et al., 2011; Nasser et al., 2013).

Y‐MHESH is a collaboration between the Universities of York (UK) and Auckland (NZ). It aims to bring together research evidence to answer questions about mental health that are important to young people. Y‐MHESH works in partnership with young people to co‐design and co‐produce meaningful evidence syntheses that have genuine impact on mental health decision‐making with, and for, young people.

Coproducing reviews and evidence synthesis in this way takes time and can lay bare differences in understanding between researchers and young people (Knowles et al., 2022). It also requires young people to develop knowledge of research methods and concepts such as ‘outcomes’ and ‘synthesis’. The apprenticeship scheme was able to support the young people to engage with this process in a deeper way than could be achieved through traditional PPI approaches.

## RESULTS

### Priority topics for evidence synthesis

The Healthy Minds Apprentices identified their three priority research questions for systematic reviews (1) first responder responses to those who self‐harm, (2) the long‐term effects of cyberbullying on mental health and (3) impacts of befriending on the mental health of those providing support.

### Output 1: Systematic review animation

**FIGURE 1:**
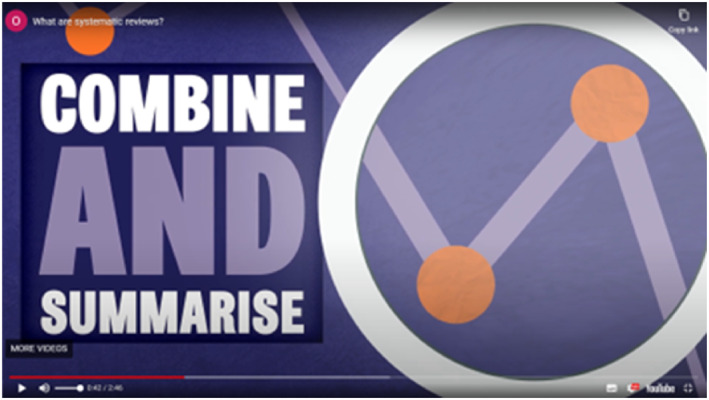
Screenshot of the short animation ‘what are systematic reviews’? Wadman & Churchill (2023). What are systematic reviews? YouTube. https://www.youtube.com/watch?v=J5PsE0vxyH4.

As a starting point for Y‐MHESH, the Healthy Minds Apprentices needed to learn about systematic reviews and evidence synthesis. We created an accessible PowerPoint presentation explaining the basics in an introductory workshop. However, the Apprentices gained a much better understanding of these terms by coproducing a short animation ‘*What are Systematic Reviews*’ (Figure 1). They draughted a script for the animation voice‐over, selected the animation style and reviewed the voice‐actors audition recordings. They emphasised the importance of getting the right voice with a regional accent and an appropriate tone. They advised us to add a content warning at the start of the animation as self‐harm is mentioned.

### Output 2: Short film ‘what is Y‐MHESH?’

**FIGURE 2:**
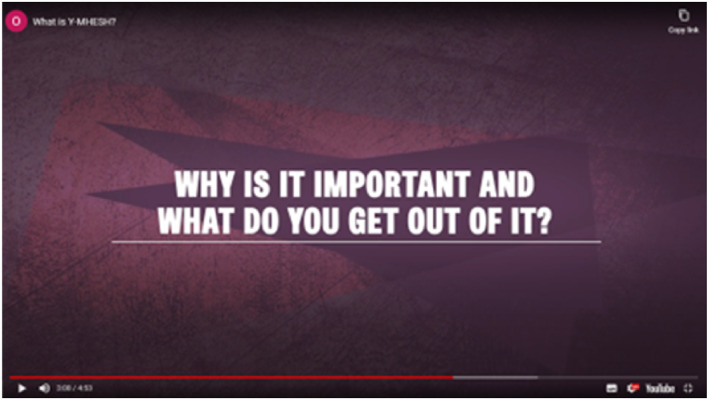
Screenshot of the Y‐MHESH short film. Churchill & Wadman (2023). What is Y‐MHESH? YouTube. https://www.youtube.com/watch?v=eH9ofB2f6eU.

We also commissioned the video production company to produce a short film with the young people, to describe Y‐MHESH and their experiences of working on the project (Figure 2). This involved two full days of filming. The researchers took it in turns to interview the Apprentices on film, asking questions including ‘*what is Y‐MHESH?*’, ‘*what is the process?*’ and ‘*how can the Healthy Minds Apprentices make a difference?*’

The Apprentices gained valuable experience in the producing the film and the animation. Some of them were particularly interested in pursuing careers in the arts and creative industries, and this opportunity allowed them to experience the behind‐the‐scenes process. They collaborated with an external media company, where they had the chance to shape the content and experience being filmed.

### Output 3: Y‐MHESH website

The Apprentices highlighted the value of having a web presence for effective engagement with different organisations and projects. The Apprentices worked with us to design a website for Y‐MHESH.

The website for Y‐MHESH was designed through an iterative process; at first the Apprentices shared ideas of webpages they liked and disliked. Using this feedback the webpages (e.g., *Home*, *About us*, *Research*) were designed and presented to the Apprentices. They gave feedback on the colour scheme, home page layout and content to be included, as well as the wording of the content. They advised us to simplify the home page and to include either the short animation or the short film at the top of the homepage, as this would draw people's attention and engages people quickly. They also provided input on the general layout which included sizes of headings, tab ordering and which images were most appropriate to use on each page. The Apprentices explored different colour scheme options and agreed the final colours. Keeping to the format and design set out by the Apprentices, the website content is currently being developed and updated to include information about the first of the priority‐questions identified by the Apprentices.

## DISCUSSION

### Advantages of working with the healthy minds apprentices

The reciprocal benefits of being able to coproduce research with an established group who were receiving specialist training in working in the mental health and youth work sectors were notable:

‐ Community engagement: The Apprentices were genuinely integrated in the local community and were able to bring learning from other projects (e.g., with schools or mental health services) and apply this to the research.
‐ Flexible working: The Apprentices set out their preferred ways of working (e.g., length of sessions, types of activities) so that our time with them was productive and enjoyable.
‐ Personal and professional development: Individual Apprentices were able to choose their level of involvement with the research, reflecting their interests, skills and career ambitions. They had the option to develop generalisable skills and also to gain academic work‐related experiences.
‐ Confidence building: The apprenticeship scheme had played an important role in building the young people's confidence. We were struck by how readily the Apprentices were able to learn about, and think critically about, new mental health topics and research methods. The Apprentices were also confident speaking to groups of academics and researchers.
‐ Mentorship: The Apprentices were situated within an existing supportive infrastructure and supervised by two project workers.
‐ Future working: we have remained in contact with three of the Apprentices either because they have moved into the role of project worker (supervising the new cohort of apprentices) or because they secured a research position locally. We hope these links will support future working together.
‐ Relationship building: The Apprentices worked together as a group on a daily basis and were familiar with each other's backgrounds, motivations, and aspirations. We felt this made it easier to establish a good working relationship with them, building on their existing relationships.

The Apprentices were employed and as such viewed their work with us as part of their professional role. It was therefore perhaps easier for them to recognise themselves as ‘professionals’ in the coproduction process and equal partners with the researchers (Mayer & McKenzie, 2017). As the Apprentices were an established group, we were able to quickly develop a partnership with them at the start of the research process and focus time on relationship building, which is recommended practice (Bailey et al., 2024; Perowne et al., 2025). They were able to acquire transferable skills and research skills, which is one of the advantages of the principle of reciprocity in coproduction (Anyon et al., 2018; Warraitch et al., 2024). As researchers, we also benefitted from the Apprentices' involvement in Y‐MHESH, particularly in learning how to explain research methodology such as evidence synthesis to young people. Our experience has also encouraged us to think about more creative and enjoyable ways to engage with young people providing them with opportunities that they would also find rewarding. Creative engagement is a recommended strategy for mental health research involvement, particular in under‐represented young people (Perowne et al., 2025). We also benefitted from the ‘local knowledge’ the Apprentices had gained through their work with services and schools (Amann & Sleigh, 2021).

Through this process we have identified two effective ways of working with young people's groups: (1) early involvement focussing first on building relationships rather than doing research (e.g., informal meetings and team building tasks) and (2) seeking regular feedback from the group and committing to act, adapt and feedback on how we have used their advice (thus recognising the value of the group's work).

For evidence syntheses, engaging young people from the earliest stages and investing in relationship‐building can create the foundation for meaningful collaboration. Early involvement enables young people to identify priorities and significantly shape the research questions, ensuring that the review focuses on issues that are relevant, timely, and aligned with the challenges they experience. Furthermore, building a feedback loop across all stages of the evidence synthesis helps demonstrate to young people how their input has influenced the process (or why certain suggestions were not adopted) and supports researchers in maintaining awareness of the wider context of their research.

### Challenges encountered

We encountered some challenges when working with the Healthy Minds Apprentices.

‐ Adapting to the group dynamic: The group dynamic changed depending on how many people were present in a workshop session. This affected how much ground we could cover in a session and also the degree to which each group member contributed to discussions. On some occasions, we sought additional feedback from individuals outside the face‐to‐face workshops.
‐ Limited availability: The Apprentices had a busy job and we would have liked more time to work with them. We were not always able to secure the meeting time we wanted and our workshops needed to be scheduled to fit around the group's main activities.
‐ Group managed by an external party: Our access to the apprentice group was necessarily managed via the organisation who employed them. There were occasions where we would have liked to have worked more with individual Apprentices whose interests most aligned with ours (e.g., to work on publications or funding applications), but this was only possible on a few occasions. Also, we did not have input into the Apprentices' personal/professional development programme, in which we ideally would have included more research training opportunities.
‐ Limited funds: We only had a small amount of funding to allow us to work with the Apprentices to set up Y‐MHESH and identify the priorities for the systematic reviews.
‐ Navigating a different payment process: Paying for young people's involvement via their salaried job was an unfamiliar process and not straightforward.

These challenges mostly align with broader discussions in the coproduction and involvement literature, such as time commitment to engage with research and lack of incentives to participate (Bailey et al., 2024; Perowne et al., 2025). To foster effective collaboration and meaningful involvement in research, it is critical to consider issues such as acceptability, accessibility, flexibility, adequate support, and any constraining factors (Reed et al., 2021; Smith et al., 2022).

We suggest drawing upon the recommendations in the ‘Kickstarting careers in research’ evaluation of a peer researcher training scheme (The Young Foundation, July 2022), in particular those addressing capacity limitations and support arrangements, recruitment challenges and designing‐in career opportunities. These recommendations include (1) ensuring sufficient resources to provide appropriate and inclusive support to young people, (2) allowing adequate time for training and onboarding young people, (3) providing opportunities for young people to collaborate on other projects with different teams, and (4) integrating career support continuously throughout the project (rather than at set points).

These four recommendations are relevant to involving young people in evidence syntheses. For instance, evidence synthesis projects do not always include sufficient resources to support advisory groups or involvement activities. In addition, given the often complex nature of mental health evidence synthesis, young people require appropriate training and ongoing support to participate effectively and confidently in the process.

### Ongoing work

At the time of writing, work has started on the first evidence synthesis priority topic working with one of the former Apprentices; a mixed methods review of emergency care following an episode of self‐harm. This was made possible by the early engagement with the Apprentices at the question formulation stage. Early involvement with young people, for example, in planning the methods or developing the search, is rare in systematic reviews but it is possible to do and can improve the relevance of the research question (Warraitch et al., 2025). We were also pleased to be able to involve another Apprentice (CP) in writing this paper (see Box 1). Website content also continues to be developed, following the guidance from the Apprentices.

**BOX 1 Reflections from a healthy minds apprentice**

As an apprenticeship team, from 2022 to 2023 we were involved in various co‐production sessions. A lot of our work was around reviewing research plans and giving our own views and opinions on young people's mental health. We advised what can be done to ensure young people's voices are heard and that both services and researchers are doing what's best for young people.
We had the opportunity to examine a range of research resources specifically designed for young people. Through this process, we provided feedback on their effectiveness and offered insights into potential improvements to enhance youth engagement. Being able to provide a balance of positive and negative views was important to us as positive feedback highlights what is effective, allowing the research team to retain and build upon successful elements of specific resources. Negative feedback, on the other hand, identified areas for improvement, helping to address weaknesses and make the resources more engaging, accessible, and relevant to the target audience. This balanced approach leads to more effective, user‐centred resources that better meet the needs of young people.
Additionally, during the co‐production sessions, our perspectives on what we believed would be beneficial for young people were clearly acknowledged and appreciated by the research team. This recognition not only reinforced the value of our contributions but also fostered a sense of trust and rapport within our apprentice team, and towards the research team. Knowing that our insights were being considered strengthened our confidence in the process and enhanced our engagement in the discussions.
As a result of the trusting relationship that was established, we felt comfortable expressing our perspectives on the challenges young people often face when engaging with researchers. We discussed the common assumption that, as young people, our views may not be fully understood or taken seriously within academic settings. However, this research team demonstrated a recognition of the value of lived experience, acknowledging it as an essential contribution alongside academic knowledge. This approach fostered a sense of respect and inclusivity, reinforcing our confidence in the co‐production process.

### Next steps

The apprenticeships came to an end in September 2023, with a new cohort being appointed. We were able to hold a celebration event for the Apprentices which included the opportunity to share reflections of working on Y‐MHESH and the premiere of the Y‐MHESH short film. Next, we believe it would be valuable to collaborate with stakeholders and young people to map existing evidence on youth mental health, doing so in ways—such as through training and evidence summaries—that support young people to engage in informed discussions and joint decision‐making to shape our research priorities and questions. Then we will work with young people to write the protocols for the reviews and to identify core outcomes. We plan to give young people the choice of different levels of involvement, depending on their interests, skills and career ambitions (e.g., workshops, reviewing documents, co‐authorship, data analysis).

### Developing the research apprenticeship model

We would like to further develop the apprenticeship model as a more equitable and sustainable approach to research involvement and coproduction with young people. There is little evidence regarding what constitutes a supportive organisational culture for youth involvement in mental health research (Jones et al., 2024). However, we have highlighted aspects of the Healthy Minds Apprentice model that clearly facilitated a reciprocal partnership and relationship building. The necessary constraints of working with an external organisation has led us to consider how this apprenticeship model could be adapted into a higher education environment.

We propose an internal university‐based apprenticeship scheme targeted at young people seeking exposure to higher education and health research. By following the recommendations in the ‘Kickstarting careers in research’ evaluation (The Young Foundation, July 2022), we will focus on establishing reciprocal working relationships to build a group of young people who are developing skills in research and interest in academic life. We want to share power and give young people ownership of this group by providing opportunities to be involved in research in diverse ways, taking on multiple roles and training. We believe this scheme sits well with the ‘public good’ remit of universities. Furthermore, this approach offers a practical and potentially scalable framework for embedding meaningful youth participation across research settings, supporting the development of transferable skills and confidence beyond the field of mental health. By promoting sustained engagement and mutual learning, the approach could also be relevant to other fields of research, such as psychology and education, where collaboration with young people can enrich both research quality and impact.

### Further research

Further research to support the development of the research apprentice model could usefully seek understand the relevant organisational and structural conditions at play, such as funding mechanisms, administrative flexibility, and leadership support and buy‐in. In addition, evaluations of coproduction within evidence synthesis projects are needed to better understand the benefits and challenges of youth involvement and to identify strategies for improving practice. Finally, we found the process of writing this reflective account of our partnership with the Apprentices valuable for recognising both strengths and challenges, and for enhancing transparency around the coproduction process. We therefore recommend that future evidence synthesis studies include reflexive case studies of their coproduction activities to build collective learning and advance best practice in the field.

## CONCLUSION

There is a need for and value in partnerships like the one we developed with the Healthy Minds Apprentices, that enabled us to benefit from their community links and to work in a bottom‐up way. The benefit of having an external organisation taking care of the Apprentices' career development, work plans and training worked well, and allowed us to focus our time on the coproduction activities. Our approach to coproduction was acceptable to the group and offered some benefits to them.

We would be interested to see if a similar apprenticeship model could operate within a university as a way to support sustainable youth involvement in mental health research. We see considerable benefits of this for recruiting and retaining young people, and supporting upskilling and career development opportunities. This approach would have challenges too, including the investment needed, buy‐in from the university and a degree of institutional flexibility. By improving the way we involve young people in research, we will also improve the research and policies that impact upon them.

## AUTHOR CONTRIBUTIONS

**Ruth Wadman**: Conceptualization; methodology; supervision; resources; project administration; investigation; writing – original draft; writing – review and editing. **Olivia Taylor**: Investigation; methodology; project administration; resources; writing – review and editing; writing – original draft. **Chloe Parekh**: Writing – original draft; writing – review and editing; resources; **Rachel Churchill**: Conceptualization; investigation; methodology; supervision; writing – review and editing.

## CONFLICT OF INTEREST STATEMENT

The authors declare no conflicts of interest.

## ETHICAL CONSIDERATIONS

An ethical approval number is not given as data is not presented in this article.

## Data Availability

Data sharing not applicable to this article as no datasets were generated or analysed during the current study.
